# Dr. Duncan S. Allen

**Published:** 1917-01

**Authors:** 


**Dr. Duncan S. Allen,** Albany 1864, died at his home in Seneca, on the night of Dec. 22-3, aged 77. He was born in Steuben Co. and came to Seneca about 1870. He was a granduncle of Dr. J. S. Allen of Geneva.

